# Identification of cuproptosis-related genes for predicting the development of prostate cancer

**DOI:** 10.1515/med-2023-0717

**Published:** 2023-09-06

**Authors:** Xin’an Wang, Xi Chen, Chengdang Xu, Weidong Zhou, Denglong Wu

**Affiliations:** Department of Urology, Tongji Hospital, School of Medicine, Tongji University, Shanghai, 200065, China; Department of Urology, Tongji Hospital, School of Medicine, Tongji University, 389, Xincun Road, Shanghai, 200065, China

**Keywords:** cuproptosis, hub gene, prostate cancer, bioinformatics analysis, cancer development

## Abstract

Copper can be toxic at very high intracellular concentrations and can inhibit prostate cancer (PCa) progression. Recently, a study reported the mechanism of cuproptosis and the potentially associated genes. However, the function of these cuproptosis-related genes in PCa remains unknown. Based on the RNA sequence and clinical data from public databases, we analyzed the clinical value of cuproptosis-related genes in PCa. *DLD*, *DLAT*, *PDHA1*, and *CDKN2A* were expressed differently between normal and PCa tissues. The *FDX1*, *LIAS*, *DLAT*, *GLS*, and *CDKN2A* genes can affect PCa progression, while *PDHA1* and *CDKN2A* influence the patients’ disease-free survival (DFS) status. The expression of *LIAS*, *LIPT1*, *DLAT*, and *PDHB* did not alter upon the incidence of PCa in Chinese patients. A constructed regression model showed that *FDX1*, *PDHA1*, *MTF1*, and *CDKN2A* can be risk factors leading to PCa in both Western and Chinese patients with PCa. The lasso regression model reflected that these genes can affect the patients’ DFS status. Additionally, the cuproptosis-related genes were associated with immune cell infiltration. We also verified the high expression of *PDHA1* and *CDKN2A*, in clinical samples. In conclusion, we identified a novel cuproptosis-related gene signature for predicting the development of PCa.

## 1 Introduction

Prostate cancer (PCa) is among the most common cancers in elderly men and showed the highest morbidity and second highest mortality rates in America in 2021 [1]. In China, both the morbidity and mortality rates of PCa have been increasing rapidly [2], seriously threatening the health of elderly men. Many factors like diet, environment, and genetic mutations and alterations can influence the occurrence of PCa [3–6]. With the development of many cancer-related genetic databases, such as The Cancer Genome Atlas (TCGA) and Prostate Cancer Genome and Epigenome Atlas (CPGEA), the specific genetic variations in PCa can be analyzed.

Regulated cell death (RCD) plays a critical role in organismal development, pathogenesis, and cancer development [7]. Several RCD types have been reported, such as apoptosis, necroptosis, and ferroptosis [8,9]. The role of RCD in PCa has been proved [10,11]. Cuproptosis is a new concept wherein the cell death process is regulated by copper [12]. The function of copper in suppressing the progression of PCa has been reported [13,14]. However, the function of cuproptosis-related genes in PCa remains unclear.

Recently, using whole-genome CRISPR-Cas9 technology, Tsvetkov et al. found ten key genes (*FDX1*, *LIAS*, *LIPT1*, *DLD*, *DLAT*, *PDHA1*, *PDHB*, *MTF1*, *GLS*, and *CDKN2A*) that are critical for cuproptosis *in vitro* [15]. Seven of these ten genes (*FDX1*, *LIPT1*, *LIAS*, *DLD*, *DLAT*, *PDHA1*, and *PDHB*) were reported to trigger cuproptosis, while the remaining three were reported to suppress cuproptosis. Further, functional analysis of these ten genes revealed that they were mainly enriched in the lipoxygenase pathway and the pyruvate dehydrogenase complexes, which are both important for tricarboxylic acid (TCA) cycle [16,17]. The role of cuproptosis-related genes in carcinomas has also been widely reported. Lei et al. reported that cuproptosis-related genes can be a prognostic biomarker for cervical cancer [18]. Bian et al. found that cuproptosis-related genes form an effective prognostic gene signature for clear cell renal cell carcinoma [19]. In addition, cuproptosis-related genes can also predict the outcome of patients with bladder cancer [20]. These studies indicate the critical role of cuproptosis-related genes in cancers.

Though many studies have reported the importance of cuproptosis-related genes in cancers, their role in PCa remains unclear. Therefore, in the current study, we attempted to determine the function of the previously reported ten cuproptosis-related genes in PCa. Using the TCGA and CPGEA databases, we analyzed these genes in PCa samples and their potential role in PCa progression and survival. We also carried out regression analysis and constructed a risk factor prediction model to predict the function of the key genes in PCa development. Potential drugs that correlated with cuproptosis-related genes and the association between cuproptosis-related genes and immune cells in PCa were also analyzed. Finally, we tested the expression of two cuproptosis-related genes, namely *PDHA1* and *CDKN2A*, in clinical samples. This study is expected to provide insights into the role of cuproptosis-related genes in PCa development.

## 2 Methods

### 2.1 Data source

The RNA sequence and clinical data of patients with PCa were obtained from the TCGA database (http://cancergenome.nih.gov/). Additionally, the data of Chinese patients with PCa were obtained from the CPGEA database (http://www.cpgea.com).

### 2.2 Data analysis

Raw RNA-sequence data downloaded from TCGA and CPGEA databases were analyzed using R software (R version 4.0.3). The primary data were normalized using the “limma” package [21]. The Series Matrix files from these two databases were mapped to the corresponding genes according to the SOFT formatted family files.

### 2.3 Online web tools

The University of Alabama at Birmingham CANcer data analysis Portal (UALCAN) (http://ualcan.path.uab.edu/), Gene Expression Profiling Interactive Analysis (GEPIA) (http://gepia.cancer-pku.cn/), Gene Set Cancer Analysis (GSCA) (http://bioinfo.life.hust.edu.cn/GSCA/), and Tumor Immune Estimation Resource (TIMER) (https://cistrome.shinyapps.io/timer/) were used in the study.

### 2.4 Functional analysis

After identifying the candidate genes, The Database for Annotation, Visualization, and Integrated Discovery v6.8 (https://david.ncifcrf.gov/) was used to perform functional analysis to identify the pathways that these genes were enriched in the study by Dennis et al. [22]. Bubble diagrams using R software were constructed to reflect the enriched pathways.

### 2.5 Mutation annotation format (MAF)

MAF is a type of mutation annotation information stored on the TCGA database. These data can aid in finding the potential mutations occurring in key genes in cancer. According to the data from the TCGA database, the mutation types of these key genes in patients with PCa were analyzed using the R software “maftools” package. Additionally, the mutation type information was also acquired from the cbioportal (https://www.cbioportal.org/) online web tool.

### 2.6 Survival analysis

The correlation between the mRNA level of cuproptosis-related genes and disease-free survival (DFS) and overall survival (OS) status of patients was analyzed using the GEPIA online web tool.

### 2.7 Expression of cuproptosis-related genes in Chinese patients with PCa

The gene expression in Chinese patients with PCa was analyzed depending on the data from the CPGEA database. The data were analyzed using the R software “limma” package and the graph was made using the “ggplot2” package.

### 2.8 Regression model

The logistic regression model can help us understand the function of key genes leading to PCa. A Lasso regression model was built to select optimal prognostic genes related to DFS. The risk score was calculated using the following formula: risk score = Σ^*j*^_*n* = 1_ Coef *j*∗*Xj*, with Coef *j* referring to the coefficient calculated by Lasso and *Xj* referring to the mRNA expression of key genes. Finally, nomogram and calibration diagram were constructed to reflect the results. The regression model was developed using the R software “autoReg” package.

### 2.9 Screening of potential small molecules that modulate the hub genes

GSCA database was used to analyze the potential drugs that can influence these hub genes and thereby be used to treat PCa. The correlation between drug sensitivity and mRNA expression was analyzed using the Genomics of Drug Sensitivity in Cancer (GDSC) and The Cancer Therapeutics Response Portal (CTRP) data.

### 2.10 Cuproptosis-related genes and immune infiltration in PCa

As immune cell infiltration plays an important role in PCa [23], we investigated the potential correlation between cuproptosis-related genes and immune cells in PCa. Using the online web tool TIMER, we analyzed the correlation between cuproptosis-related genes and immune cells in PCa.

### 2.11 Clinical specimen collection

PCa samples and para-cancerous samples were collected at Tongji Hospital, School of Medicine, Tongji University. The sample collection method was approved by the Ethics Committee of Tongji Hospital, School of Medicine, Tongji University (SBKT-2021-220). Informed consent was obtained from all patients who provided samples.

### 2.12 Immunohistochemistry (IHC)

The expression of PDHA1 and CDKN2A in clinical specimens was detected using IHC. Tumor samples were fixed with formalin and embedded into paraffin. Four-micrometer thick sections were cut from the samples and fixed. Thereafter, antigen retrieval and immunostaining were performed as described previously [24]. Primary antibodies against PDHA1 (Catalog No. 168379) and CDKN2A (Catalog No. 17878) were purchased from Abcam (Cambridge, UK). The secondary antibody (Catalog No. A0216) was purchased from Beyotime Biotechnology Company (Shanghai, China). The IHC score was evaluated by two independent professional pathologists.

### 2.13 Statistical analysis

All data are represented as the mean ± standard deviation (SD) obtained from at least three repeated experiments. Data were compared using Student’s *t*-test for two groups and one-way analysis of variance for three or more groups. A *P* value < 0.05 was considered to be statistically significant.


**Ethics approval:** The study was approved by the ethics committee of Tongji Hospital, School of Medicine, Tongji University (SBKT-2021-220). Each participant volunteered to join and signed the informed consent form.

## 3 Results

### 3.1 Cuproptosis-related gene expression in patients with PCa

**Figure 1:**
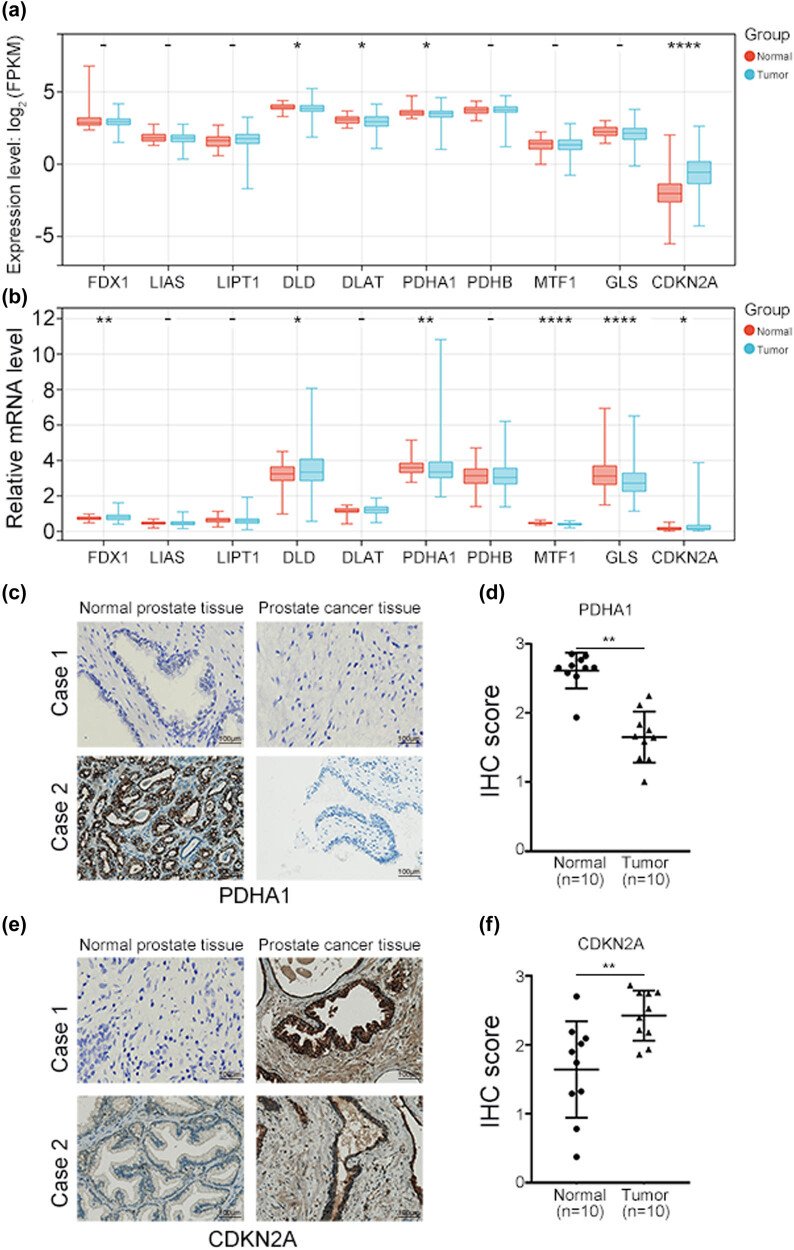
Expression of ten cuproptosis-related genes in patients with PCa from (a) TCGA database and (b) CPGEA database. (c and e) Protein level of (c) PDHA1 and (e) CDKN2A in clinical PCa tissues detected using IHC. (d and f) IHC score of (d) PDHA1 and (f) CDKN2A – represents no statistical differences, *P < 0.05, **P < 0.01, ****P < 0.001.

Based on a previous study [15], we analyzed the expression of ten cuproptosis-related genes – *FDX1*, *LIAS*, *LIPT1*, *DLD*, *DLAT*, *PDHA1*, *PDHB*, *MTF1*, *GLS*, and *CDKN2A* – in patients with PCa by downloading their RNA-sequence data from the TCGA database. We found that *DLD*, *DLAT*, *PDHA1*, and *CDKN2A* were differentially expressed between normal and tumor tissues; *CDKN2A* was upregulated and the other genes were downregulated in the tumor tissues (Figure 1a). This suggests that these genes may be critical for PCa occurrence in Western patients with PCa. As the patients’ data from the TCGA database mainly included Western populations, we attempted to verify the expression of these cuproptosis-related genes in patients of a different race by analyzing the RNA-sequence data of Chinese patients with PCa from the CPGEA database. We found that *FDX1*, *DLD*, *PDHA1*, *MTF1*, *GLS*, and *CDKN2A* showed differential expression levels between normal and tumor tissues. *FDX1*, *DLD*, and *CDKN2A* were upregulated, and *PDHA1*, *MTF1*, and *GLS* were downregulated upon the incidence of PCa, which suggests that these genes may be important for PCa occurrence in Chinese patients (Figure 1b). As *PDHA1* and *CDKN2A* showed similar differential expression patterns in both TCGA and CPGEA databases, we chose these two genes for IHC analysis in ten paired clinical samples. We found that PDHA1 was downregulated (Figure 1c and d) and CDKN2A was upregulated (Figure 1e and f) in the tumor samples. As methylation is an important gene modification process and plays an important role in PCa [25], we examined the methylation level of the cuproptosis-related genes in PCa using UALCAN. We found that except for *DLD*, *PDHA1*, and *GLS*, the methylation levels of the other genes were changed in PCa. The methylation level of *DLAT*, *PDHB*, and *CDKN2A* was increased, while that of *FDX1*, *LIAS*, *LIPT1*, and *MTF1* was decreased upon the incidence of PCa (Figure S1).

### 3.2 Mutation and function analysis

**Figure 2:**
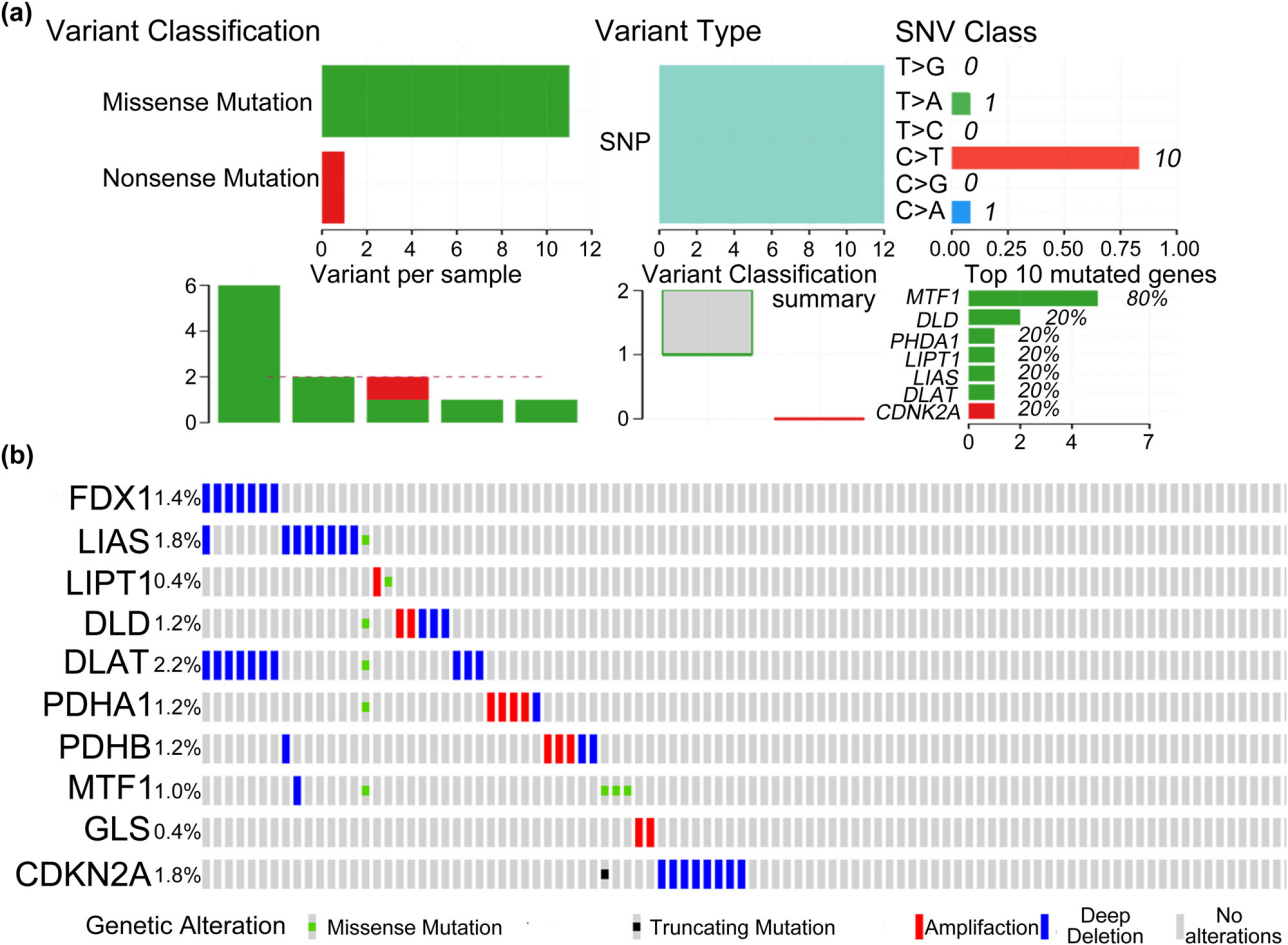
Mutation of cuproptosis-related genes in PCa. (a) MAF map of cuproptosis-related genes in PCa (data from the TCGA database). (b) Mutation rate of cuproptosis-related genes in PCa (data from cbioportal online web tool).

As the candidate cuproptosis-related genes are important for the occurrence of PCa, we next analyzed the potential mutation types of these genes in PCa. Using the mutation information from the TCGA database, we carried out an MAF analysis. We found that the most frequent variant type is single nucleotide polymorphism, and *MTF1* and *DLD* are the top two genes whose mutations are associated with PCa occurrence (Figure 2a). Next, we used the online web tool cbioportal to verify the results and found that *DLAT*, *LIAS*, and *CDKN2A* have the highest probability of a mutation occurring in the gene (Figure 2b). Next, we analyzed the pathways in which these cuproptosis-related genes were enriched via GO annotation and KEGG pathway analyses. Both analyses revealed that these cuproptosis-related genes are mainly enriched in the acetyl-CoA biosynthesis process (Figure S2a) and TCA cycle (Figure S2b), which coincided with the results previously found [15].

### 3.3 Cuproptosis-related genes affect PCa progression

**Figure 3:**
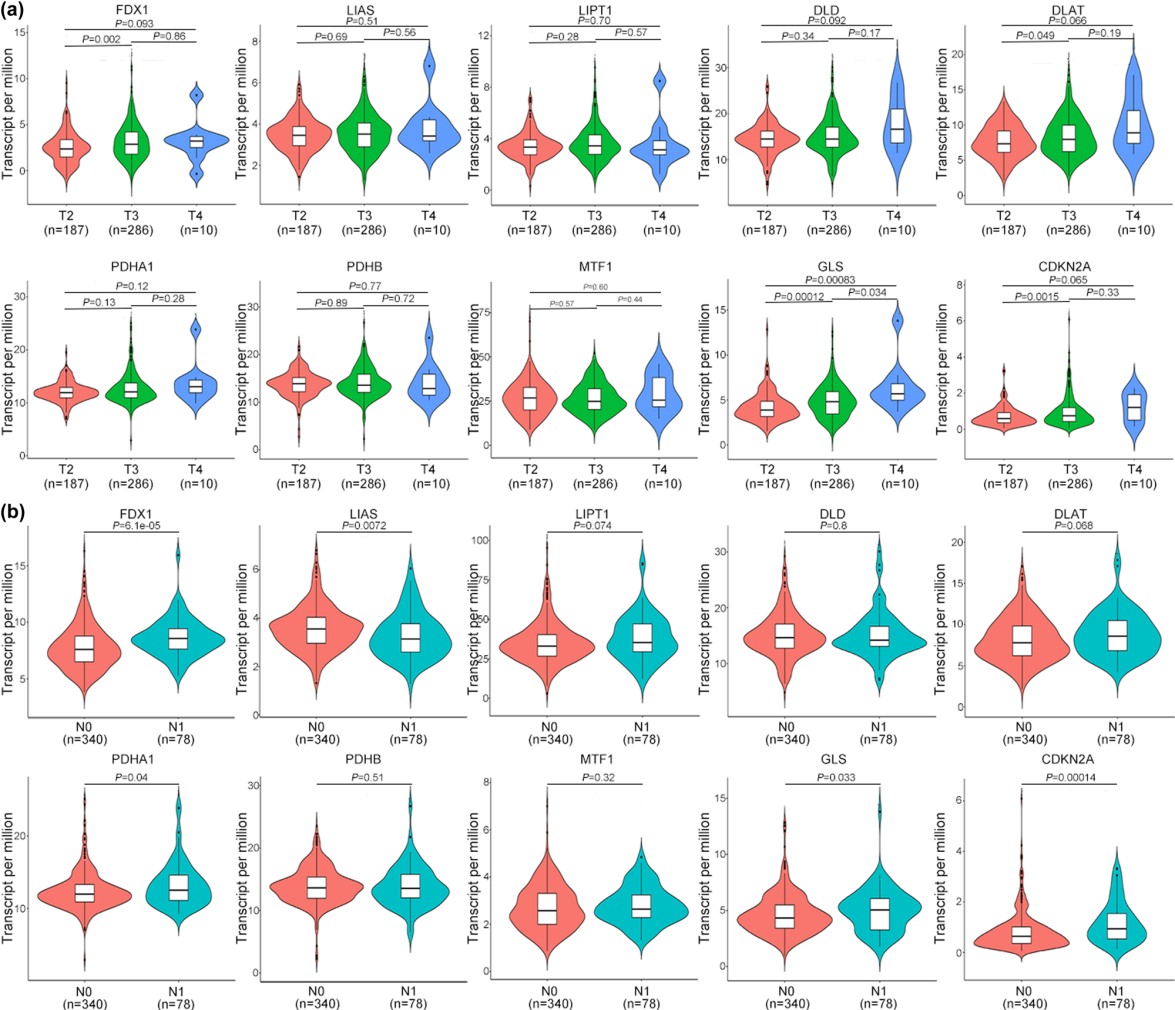
Expression of the ten cuproptosis-related genes in patients with PCa with different TNM tumor stages: (a) T stage and (b) N stage.

Next, we tested whether, in addition to PCa occurrence, these cuproptosis-related genes can affect PCa progression. Tumor–node–metastasis (TNM) classification of malignant tumors is commonly used to score the severity of PCa [26]. Therefore, we analyzed the expression of these key genes in different TNM stages of PCa depending on the TCGA data. As a sufficient number of patients did not show metastasis, we did not analyze the metastasis (M) stage. In this analysis, we found that the expression of *FDX1*, *GLS*, and *CDKN2A* increased when the tumor progressed from the T2 stage to the T3 stage. The expression of *GLS* also increased when the tumor progressed from the T3 to the T4 stage (Figure 3a). These results indicated that some of these cuproptosis-related genes can affect PCa primary tumor progression. The node–metastasis (N) stage was also considered in this study. We found that the expression of *FDX1*, *PDHA1*, *GLS*, and *CDKN2A* increased upon the occurrence of lymphatic metastasis, but the expression of *LIAS* decreased post-lymphatic metastasis (Figure 3b). These results indicated that cuproptosis-related genes can affect PCa progression.

### 3.4 Risk prediction model of cuproptosis-related genes leading to PCa

**Figure 4:**
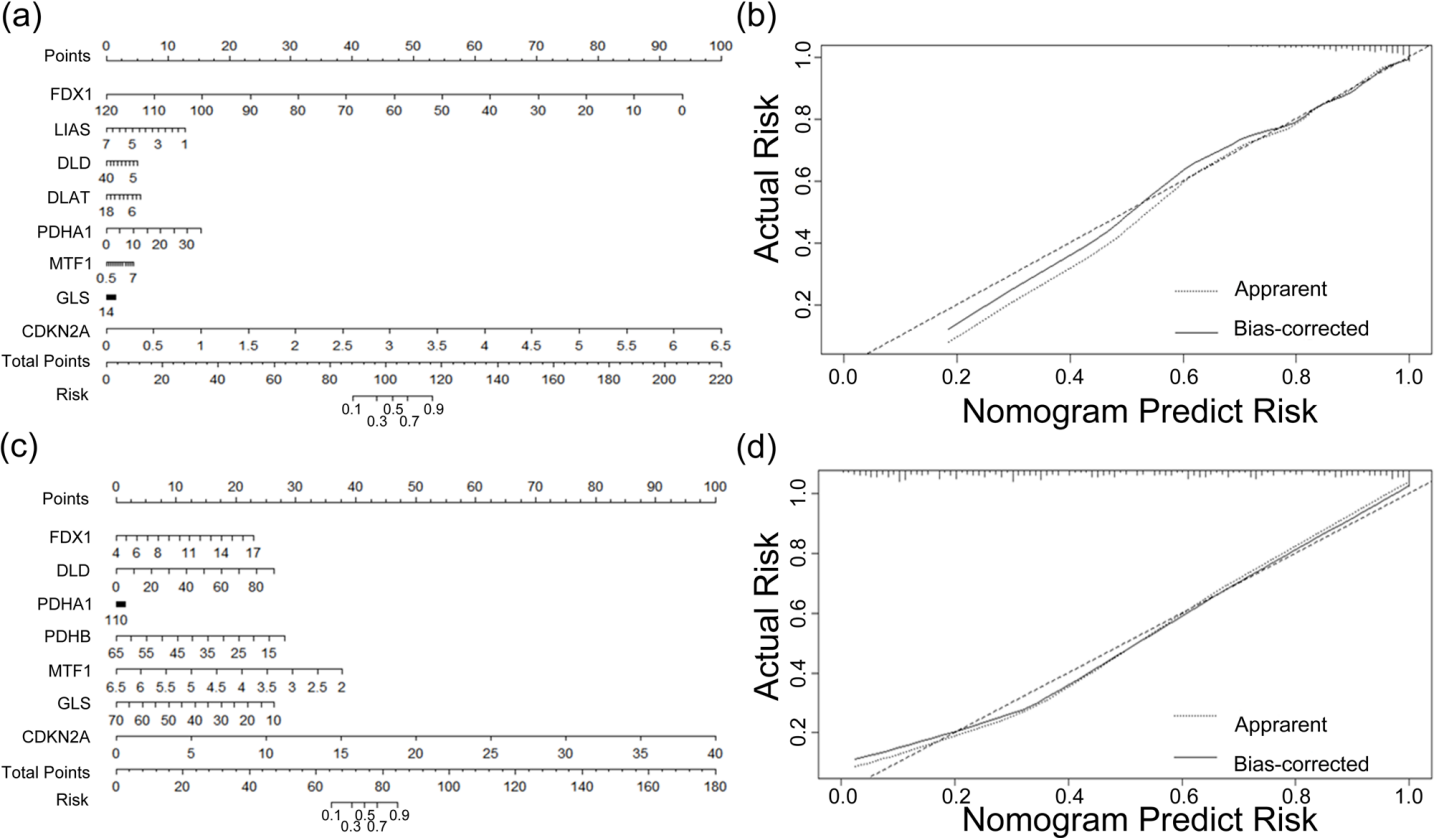
Nomogram of the logistic regression model of the value of ten cuproptosis-related genes in causing PCa. (a) Nomogram reflects the risk of ten cuproptosis-related genes in PCa occurrence on TCGA database and (b) calibration curve of the nomogram. (c) Nomogram reflecting the risk of ten cuproptosis-related genes leading to PCa on the CPGEA database and (d) calibration curve of the nomogram.

Next, we examined whether these cuproptosis-related genes can be risk factors for PCa. Depending on the data from both TCGA and CPGEA databases, we carried out logistic regression analysis to determine the risk value of these genes for PCa occurrence and built a nomogram. Using the TCGA data, we found that *FDX1* and *CDKN2A* are the top two risk genes associated with PCa occurrence (Figure 4a, nomogram in Figure 4b). Using data from the CPGEA database, we found that *MTF1* and *CDKN2A* are the top two genes associated with PCa occurrence (Figure 4c, nomogram in Figure 4d). The risk degree of cuproptosis-related genes causing PCa in both TCGA and CPGEA datasets is shown in a forest map, which indicated that *CDNK2A* is the highest-risk gene for PCa in both databases (Figure S3).

### 3.5 Prognostic value of cuproptosis-related genes in patients with PCa

**Figure 5:**
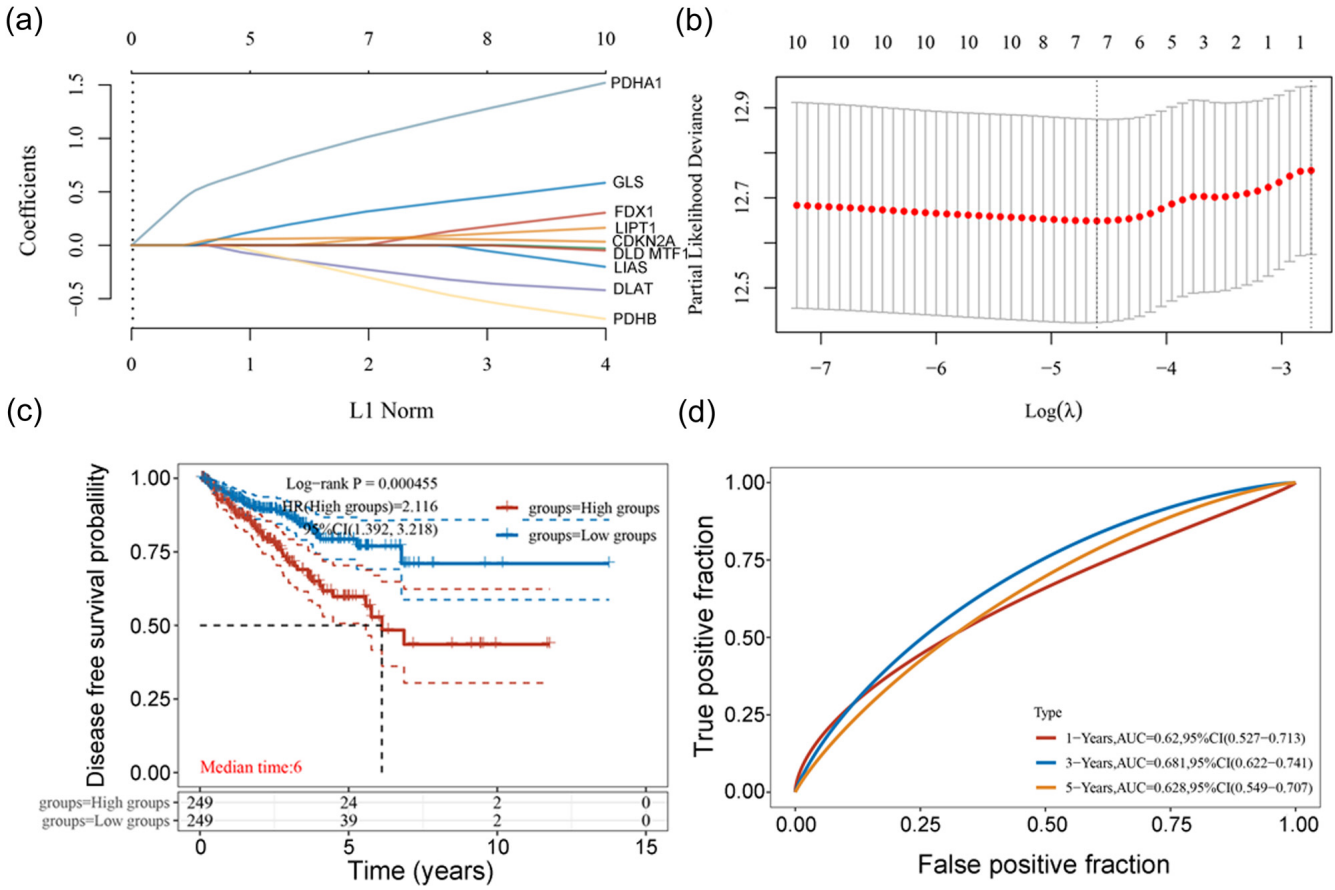
A prognostic model of cuproptosis-related genes in affecting the DFS of patients with PCa (data from TCGA database). (a) Ten-time cross-validation for tuning parameter selection in the Lasso cox regression model. (b) Lasso coefficient profiles. (c) KM curve reflects the influence of the ten cuproptosis-related genes on the DFS of the patients. (d) Time-dependent ROC curve reflecting the accuracy of the lasso model.

As the identified cuproptosis-related genes can influence PCa occurrence and progression, we tested whether they can also affect PCa prognosis. As patients with PCa have a long survival period and because there are insufficient data on the death of such patients in the TCGA database, we only tested the association of the cuproptosis-related genes with PCa DFS. We constructed a lasso regression model built to select the optimal prognostic genes related to DFS (Figure 5a and b). A total of seven genes were identified and selected to develop a prognostic signature. The risk score was calculated as follows: risk score = (0.0619 × expression of FDX1) + (0.0743 × expression of LIAS) + (−0.2705 × expression of DLAT) + (1.0965 × expression of PDHA1) + (−0.3756 × expression of PDHB) + (0.3598 × expression of GLS) + (0.065 × expression of CDKN2A). We carried out a Kaplan–Meier (KM) survival analysis of the risk model from the dataset and compared different groups using the log-rank test. In KM curve analysis, we found that patients with lower expression of these genes were associated with a longer DFS period (Figure 5c). Finally, a time-dependent receiver operating characteristic (ROC) curve was built to reflect the accuracy of the model we built. The ROC curve indicated that the built model has a preferable predictive power (Figure 5d).

### 3.6 Effect of the cuproptosis-related genes on the survival status of patients with PCa

**Figure 6:**
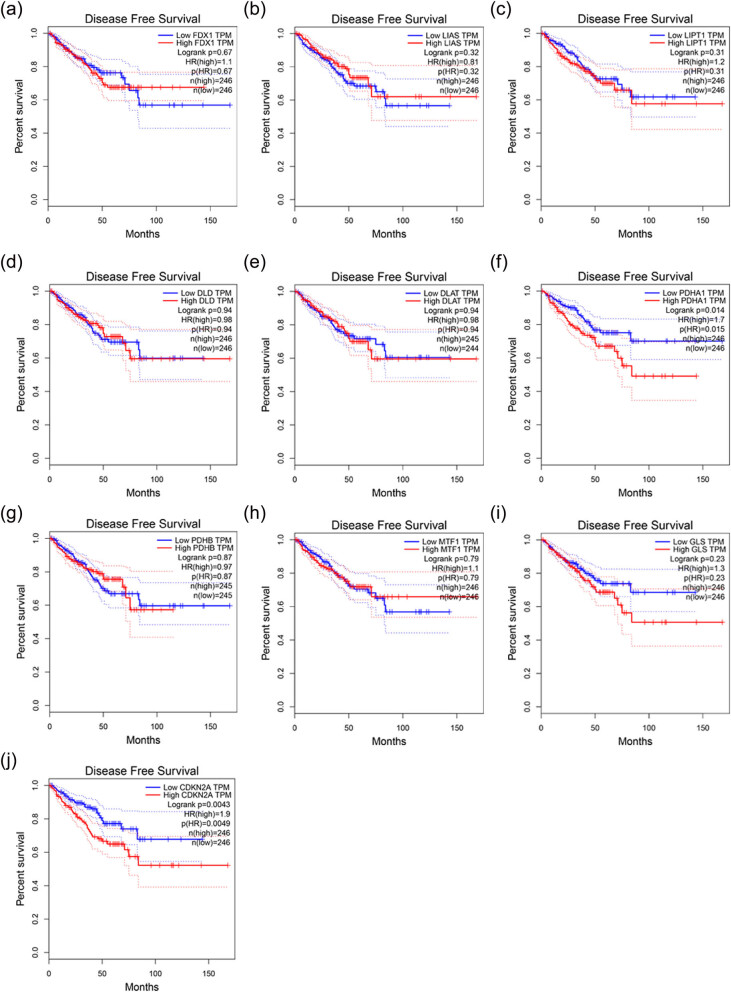
Correlation between cuproptosis-related genes and the prognosis of PCa based on the DFS status obtained from the GEPIA online tool: (a) *FDX1*, (b) *LIAS*, (c) *LIPT1*, (d) *DLD*, (e) *DLAT*, (f) *PDHA1*, (g) *PDHB*, (h) *MTF1*, (i) *GLS*, and (j) *CDKN2A*.

To determine whether the key genes can affect the survival status of patients with PCa, both DFS and OS were included in the correlation analysis using the online web tool, GEPIA. The analysis indicated that *PDHA1* and *CDKN2A* can affect the patients’ DFS status (Figure 6). However, all the tested genes were not correlated with the patients’ OS (Figure S4).

### 3.7 Potential drug influencing cuproptosis-related gene expression in PCa

**Figure 7:**
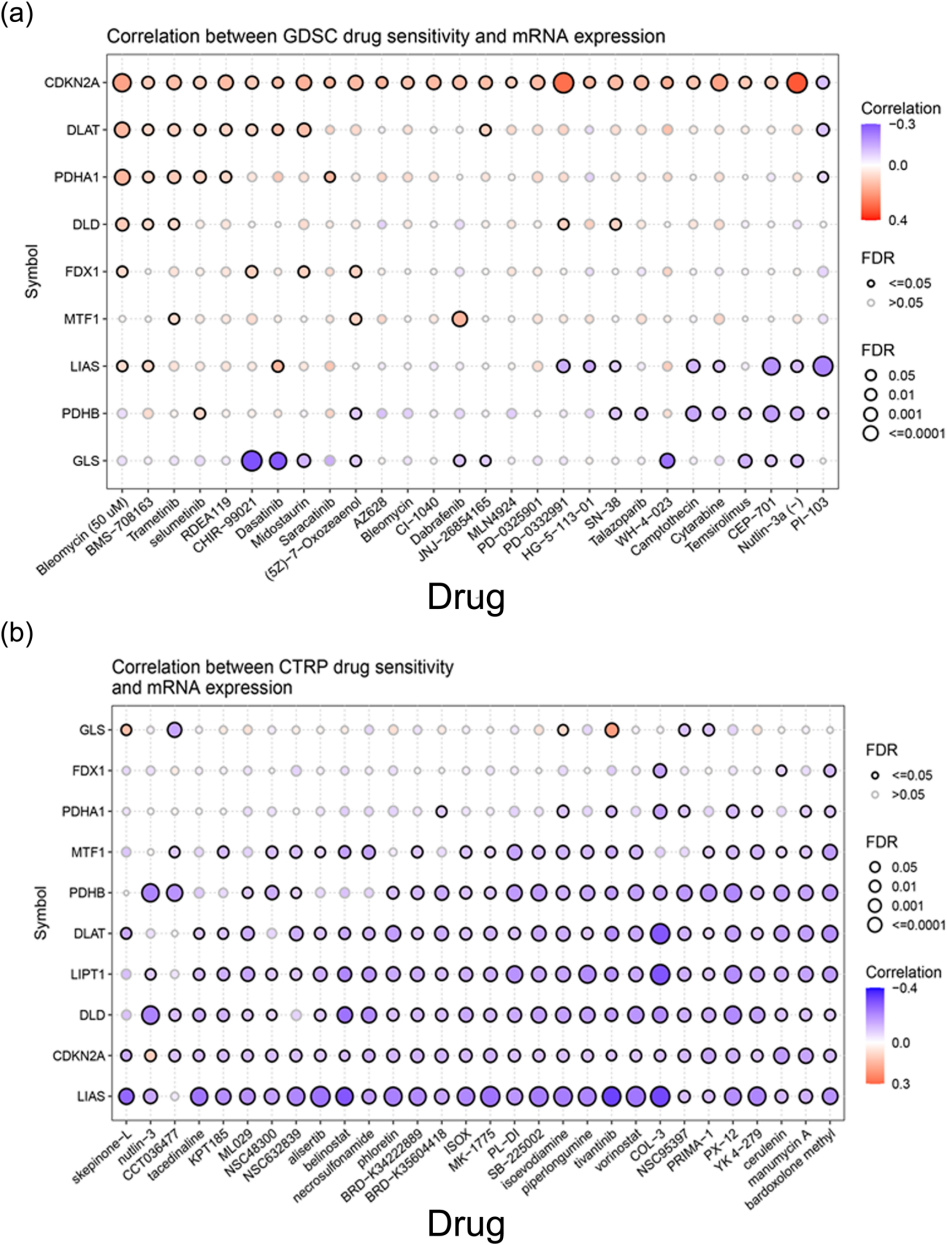
Potential drugs that can affect the expression of cuproptosis-related genes: (a) small molecules obtained from the GDSC database and (b) potential drugs obtained from the CTRP database.

Next, we tried to find potential small molecules that can influence the expression of cuproptosis-related genes and thereby be useful to treat PCa. Depending on an online web tool, GSCA, we analyzed potential drugs for treating PCa from GDSC and GTRP databases. In the GDSC database, except for *LIPT1*, other genes could be potential targets for PCa treatment (Figure 7a). In GTRP, we found that all ten genes could be potential therapeutic targets for PCa (Figure 7b). These results indicated that cuproptosis-related genes can be potential therapeutic targets for treating PCa.

### 3.8 Correlation between expression of cuproptosis-related genes and immune infiltration in PCa

**Figure 8:**
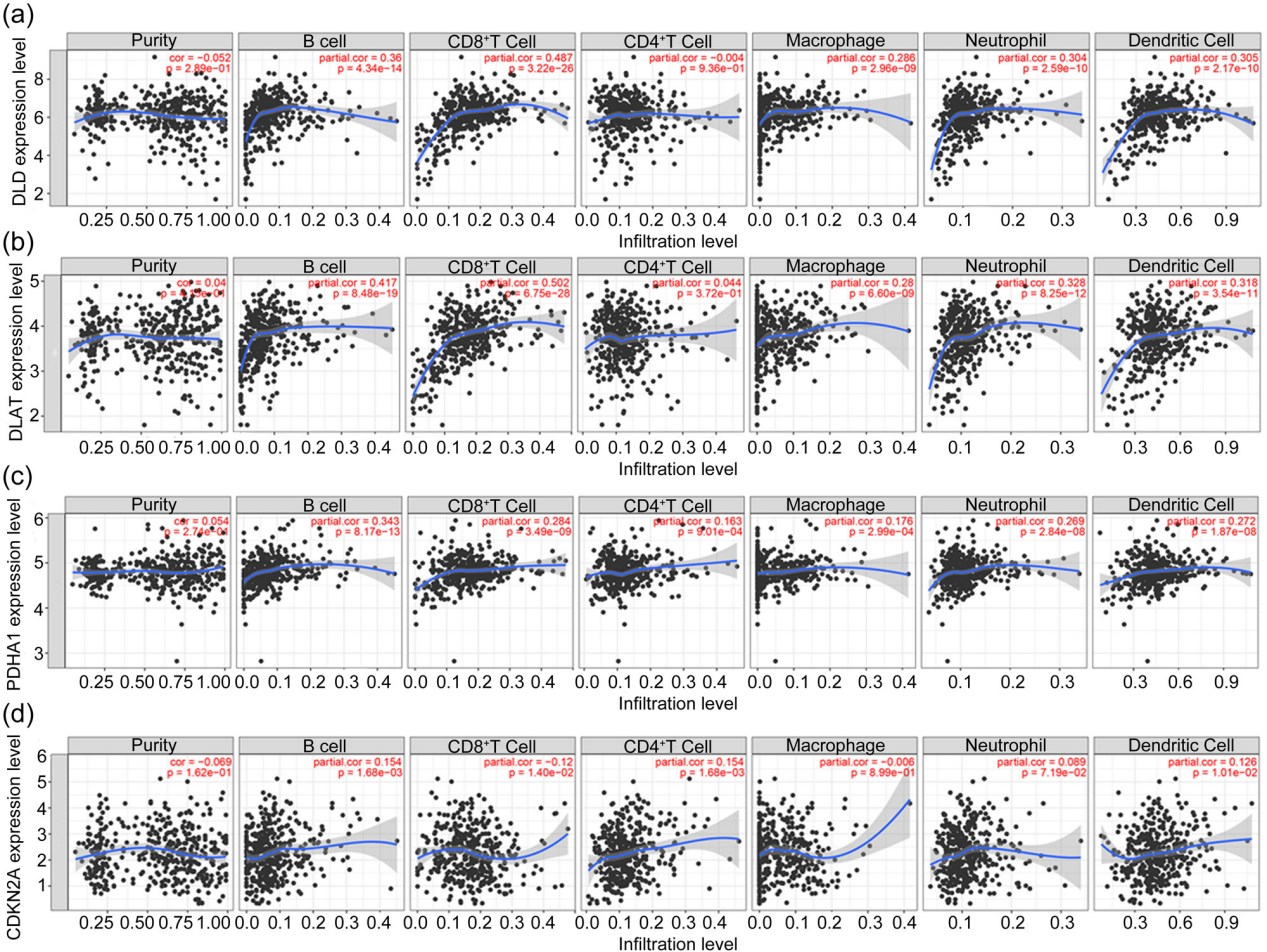
Correlation between cuproptosis-related genes and immune cells in PCa (data from TIMER online web tool): (a) *DLD*, (b) *DLAT*, (c) *PDHA1*, and (d) *CDKN2A*.

Immune cell infiltration has been reported to play an important role in PCa. In addition, several studies have proved that cuproptosis-related genes can affect immune cell infiltration and thereby the prognosis of many cancer types, including bladder cancer, gliomas, and hepatocellular carcinoma [20,27,28]. This suggests that cuproptosis-related genes may also be associated with immune cells in PCa. Therefore, we evaluated the correlation between the mRNA level of the cuproptosis-related genes and immune cells using the TIMER online web tool. Four differently-expressed cuproptosis-related genes from the TCGA database were included in the study. We found that except for Purity and CD4^+^ T cells, *DLD* showed a correlation with other immune cells in PCa (Figure 8a). *DLAT* was not associated with Purity and CD4^+^ T cell in PCa (Figure 8b). Except for Purity, other immune cells were correlated with *PDHA1* in PCa (Figure 8c). B cells, CD4^+^ T cells, CD8^+^ T cells, neutrophil cells, and dendritic cells were correlated with *CDKN2A* (Figure 8d). These results indicated that these genes may influence PCa development through immune cell infiltration.

## 4 Discussion

Both genetic and heredity factors play a central role in the occurrence and progression of cancers including PCa [4,6,29]. Zheng et al. have reported that epigenetic changes may increase the risk of breast cancer [30]. Thus, genetic changes are important for carcinoma. With the development of bioinformatics, systemic analysis of potential gene mutations has been widely used for determining the causative factors of disease occurrence. Microarray technology and bioinformatics analysis have also been used to identify gene alterations in the occurrence of PCa [31]. In this study, we verified the function of previously reported ten cuproptosis-related genes in PCa. We found that these cuproptosis-related genes play a critical role in the occurrence and development of PCa. We also found that the cuproptosis-related genes are associated with immune cell infiltration and may be potential targets for treating PCa.

Copper is an important cofactor that maintains the activity of enzymes [32]. However, a high level of copper can induce cell death [33,34]. Furthermore, genetic variation in copper homeostasis results in life-threatening diseases like Alzheimer’s, Parkinson’s, and Wilson’s disease [35,36], suggesting that copper is important in the occurrence of several diseases. The function of copper in PCa has also been reported, with studies showing that copper can affect PCa progression and drug resistance [10]. In addition, copper can be used in the diagnosis of PCa [13,37]. Another study reported that copper can inhibit PCa cell proliferation and lead to PCa cell death [38]. These studies all proved that copper is important in PCa. Recently, a study reported the mechanism of copper-induced cell death and analyzed the associated potential genes [15]. In the study, they put forward a new type of cell death named cuproptosis, which is different from the traditionally known apoptosis, necroptosis, and ferroptosis. Furthermore, based on whole-genome CRISPR-Cas9 analysis, they reported ten important genes associated with cuproptosis [15]. However, the function of these specific cuproptosis-related genes in PCa is unclear.

In this study, we found that the expression of two cuproptosis-related genes, *FDHA1* and *CDKN2A*, changed upon the occurrence of PCa. These genes also showed different expression levels in patients with PCa based on the TNM tumor stage and could influence the patients’ DFS. Further, we found that *FDHA1* was downregulated and *CDKN2A* was upregulated in clinical tumor samples, which was consistent with the results from the public databases. These findings indicated that *FDHA1* and *CDKN2A* may affect PCa progression and prognosis.

Pyruvate dehydrogenase E1 subunit alpha 1 (PDHA1), one of the multiple enzymes of the pyruvate dehydrogenase complex, catalyzes the reaction that produces acetyl-CoA and CO_2_ from pyruvate, which links glycolysis and the TCA [39]. Though the function of PDHA1 in PCa has not been reported, it can impact the occurrence of many cancers such as ovarian cancer, gastric cancer, and breast cancer by influencing glucose metabolism [40–42]. The cyclin-dependent kinase inhibitor 2A (CDNK2A) gene is located on chromosome 9p21 and has three exons that encode for the tumor suppressor protein p16. CDNK2A is frequently mutated or deleted in various tumors [43–45]. CDKN2A is correlated with many cancers such as bladder cancer, colorectal cancer, and breast cancer [46–48]. CDKN2A has also been reported to affect the survival status of patients with PCa [49]. These findings are substantiated by our results, which indicate that these two genes are important for PCa development.

This study has some limitations. First, the cuproptosis-related genes included in the study were those identified in a previous study, and there is no systematic report on cuproptosis-related genes. Hence, the included cuproptosis-related genes may not be comprehensive or representative. Second, we only analyzed the association of the expression of these cuproptosis-related genes with PCa occurrence, progression, and prognosis. However, the mechanism of these genes in PCa remains unclear. Thus, future studies should focus on investigating the specific function of these cuproptosis-related genes in PCa. Third, though we verified the expression of two cuproptosis-related genes in clinical samples, the sample size was small, which might have led to bias. Hence, our results need to be verified using more samples. Finally, we only tested the expression of two cuproptosis-related genes, namely *PDHA1* and *CDKN2A*, in clinical samples. Other cuproptosis-related genes may also be important in PCa, and this needs further investigation. Nonetheless, this is the first study to evaluate the function of cuproptosis-related genes in PCa and show that two cuproptosis-related genes, *PDHA1* and *CDKN2A*, are important in PCa occurrence, progression, and prognosis.

## 5 Conclusions

In conclusion, we systematically analyzed the function of ten cuproptosis-related genes in PCa and found two genes, *PDHA1* and *CDKN2A*, to play important roles in PCa development.

## Supplementary Material

Supplementary Figure
