# Supplementary material for: Identification of cuproptosis-related genes for predicting the development of prostate cancer
Source: Open Med (Wars). 2023 Sep 6;18(1):20230717. doi: 10.1515/med-2023-0717 (PMC10499014; doi:10.1515/med-2023-0717)
Supplement: Supplementary Figure [file med-2023-0717-sm.pdf]

Supplementary material

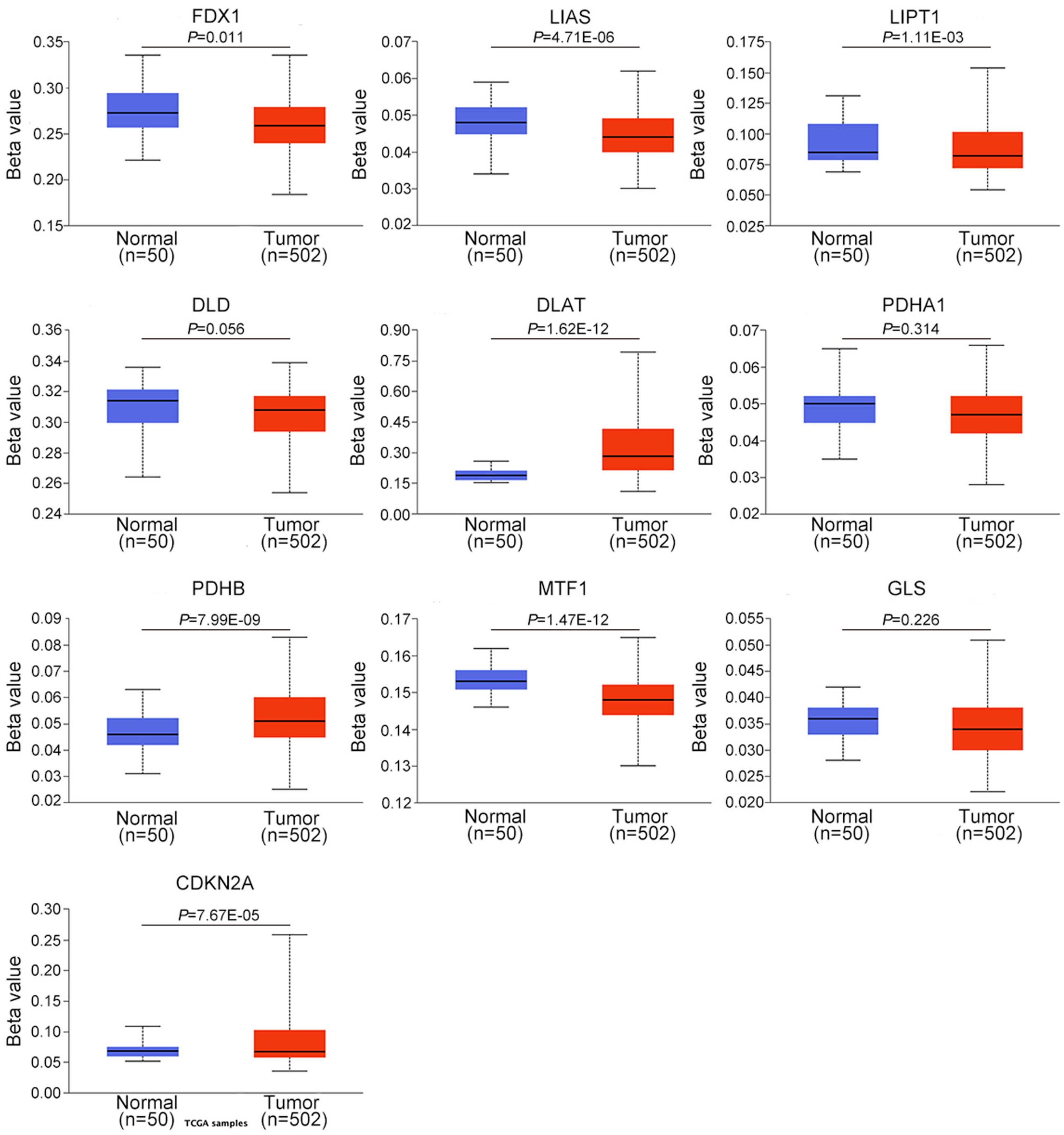

Figure S1: The methylation level of ten cuproptosis-related genes in patients with prostate cancer (PCa) (data from UALCAN online web tool).

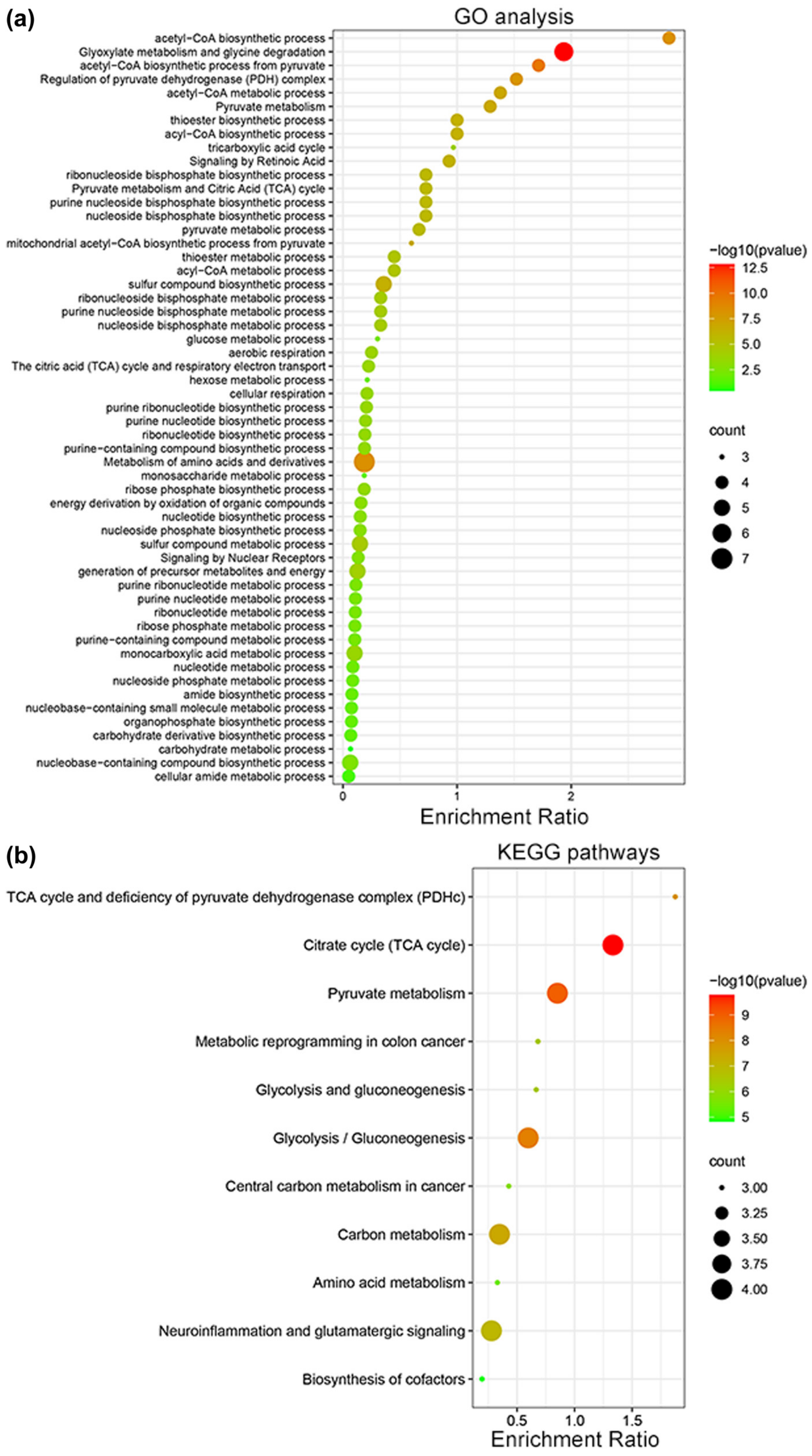

Figure S2: The pathways of cuproptosis-related genes enriched. (a) The GO analysis pathways are shown. (b) The KEGG pathways are shown.

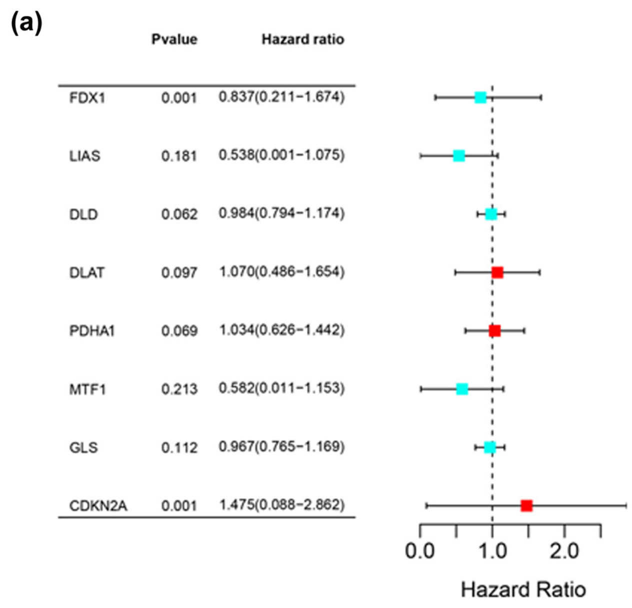

Figure S3: Forest map reflecting the risk of cuproptosis-related genes in causing prostate cancer (PCa) depending on the data from the TCGA (a) and CPGEA (b) databases.

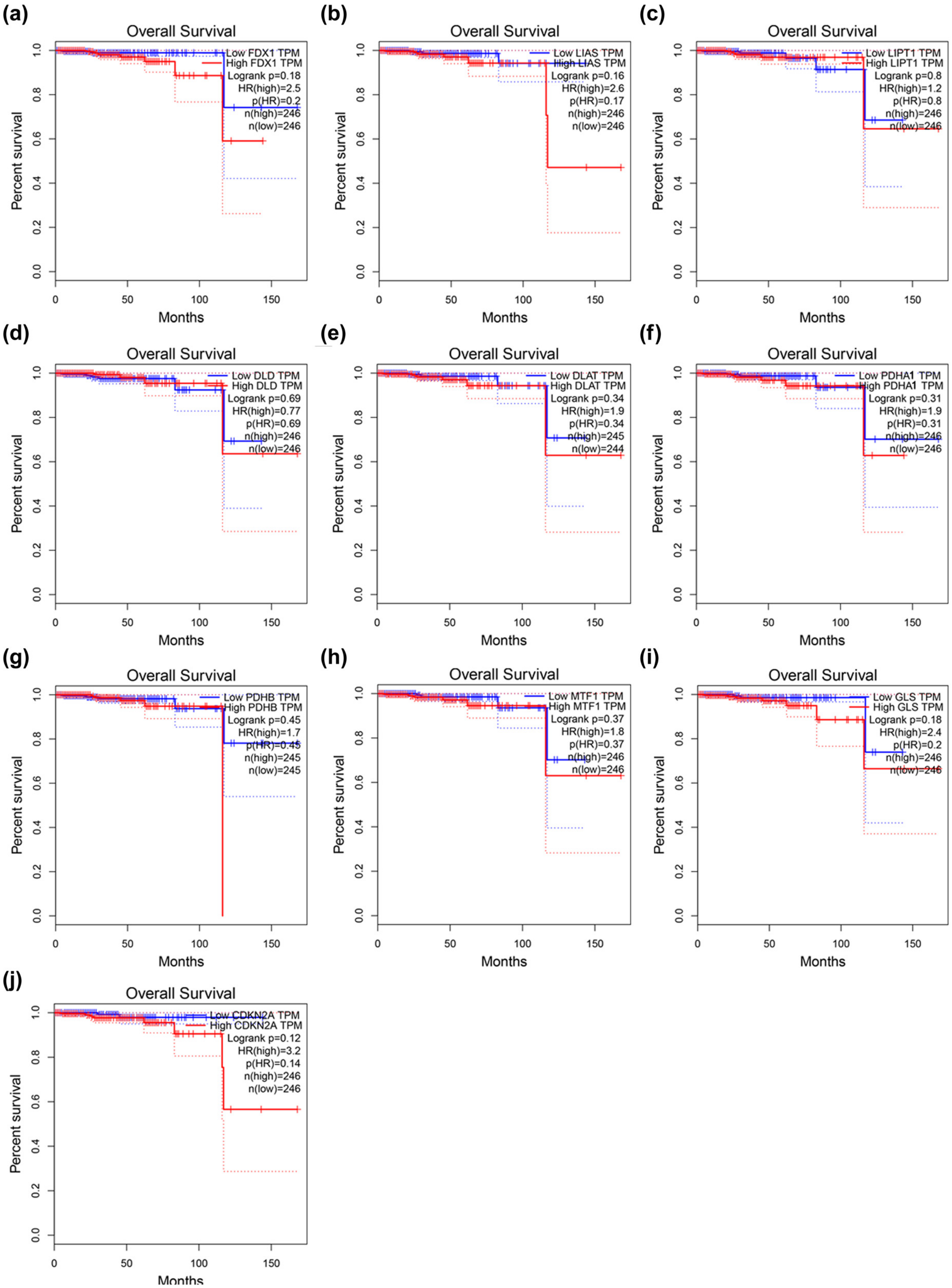

Figure S4: The correlation between cuproptosis-related genes and the prognosis of prostate cancer (PCa) with regard to the overall survival (OS) status obtained from the GEPIA online tool.
